# Study of the relation between body weight and functional limitations and pain in patients with knee osteoarthritis

**DOI:** 10.1590/S1679-45082017AO4082

**Published:** 2017

**Authors:** Fábio Marcon Alfieri, Natália Cristina de Oliveira Vargas e Silva, Linamara Rizzo Battistella

**Affiliations:** 1 Centro Universitário Adventista de São Paulo, São Paulo, SP, Brazil.; 2 Universidade de São Paulo, São Paulo, SP, Brazil.

**Keywords:** Knee joint/pathology, Osteoarthritis, knee/complications, Body weight, Body mass index, Pain, Adult, Aged, Articulação do joelho/patologia, Osteoartrite do joelho/complicações, Peso corporal, Índice de massa corporal, Dor, Adulto, Idoso

## Abstract

**Objective:**

To assess the influence of the body weight in functional capacity and pain of adult and elderly individuals with knee osteoarthritis.

**Methods:**

The sample consisted of 107 adult and elderly patients with knee osteoarthritis divided into two groups (adequate weight/adiposity and excessive weight/adiposity) according to body mass index and percent of body fat mass, assessed by electric bioimpedance. Subjects were evaluated for functional mobility (Timed Up and Go Test), pain, stiffness and function (Western Ontario and MacMaster Universities Osteoarthritis Index − WOMAC), pain intensity (Visual Analogue Scale − VAS) and pressure pain tolerance threshold (algometry in vastus medialis and vastus lateralis muscles). Data were analyzed with Statistical Package of the Social Sciences, version 22 for Windows. Comparisons between groups were made through Student’s *t* test, with significance level set at 5%.

**Results:**

There was predominance of females in the sample (81.3%), and mean age was 61.8±10.1 years. When dividing the sample by both body mass index and adiposity, 89.7% of them had weight/adiposity excess, and 59.8% were obese. There was no difference between groups regarding age, pain intensity, pressure pain tolerance threshold, functional mobility, stiffness and function. However, pain (WOMAC) was higher (p=0.05) in the group of patients with weight or adiposity excess, and pain perception according to VAS was worse in the group of obese patients (p=0.05).

**Conclusion:**

Excessive weight had negative impact in patients with osteoarthritis, increasing pain assessed by WOMAC or VAS, although no differences were observed in functionality and pressure pain tolerance.

## RESUMO

**Objetivo:**

Avaliar a influência do peso corporal na capacidade funcional e na dor de adultos e idosos com osteoartrite de joelho.

**Métodos:**

A amostra foi constituída por 107 pacientes adultos e idosos com osteoartrite do joelho, divididos em dois grupos (peso/adiposidade adequados e peso/adiposidade em excesso), de acordo com o índice de massa corporal e a porcentagem de adiposidade corporal, e avaliados por bioimpedância elétrica. Os sujeitos foram avaliados quanto à mobilidade funcional (*Timed Up and Go*), dor, rigidez e função (*Western Ontario and McMaster Universities Osteoarthritis Index* − WOMAC), intensidade da dor (Escala Visual Analógica - EVA) e limiar de limiar de dor à pressão (algometria nos músculos vasto medial e vasto lateral). Os dados foram analisados pelo *Statistical Package of the Social Sciences*, versão 22 para Windows. As comparações entre os grupos foram feitas por meio do teste *t* de Student, com nível de significância estabelecido em 5%.

**Resultados:**

Houve predomínio de mulheres na amostra (81,3%), com média de idade de 61,8±10,1 anos. Ao dividir a amostra por índice de massa corporal e adiposidade, 89,7% apresentaram peso/adiposidade em excesso, e 59,8% eram obesos. Não houve diferença entre os grupos quanto a idade, intensidade da dor, limiar de dor à pressão, mobilidade funcional, rigidez e função. Entretanto, a dor (WOMAC) foi maior (p=0,05) no grupo de pacientes com excesso de peso ou adiposidade, e a percepção da dor pela EVA foi pior no grupo de pacientes obesos (p=0,05).

**Conclusão:**

O peso em excesso teve impacto negativo em pacientes com osteoartrite, aumentando a dor avaliada pelo WOMAC ou pela EVA, embora não tenham sido observadas diferenças na funcionalidade e na tolerância à dor sob pressão.

## INTRODUCTION

Osteoarthritis (OA), one of the most prevalent chronic diseases worldwide, is the most common articular disorder and affects 50% or more elderly individuals.^(^ ^1^ ^,^ ^2^ ^)^ It impairs the knees of around 10% of women and 13% of men above 60 years of age. The proportion of symptomatic individuals is increasing due to population ageing and a rise in obesity rates in many countries.^(^ ^3^ ^)^

Osteoarthritis is the main cause of pain, incapacity and decreased quality of life of the elderly.^(^ ^4^ ^-^ ^6^ ^)^ Among the etiological factors of the disease, increased body weight must be highlighted since it causes more articular pressure, contributing to cartilage degeneration, sclerosis of the subchondral bone and formation of osteophytes.^(^ ^7^ ^,^ ^8^ ^)^ This probably explains the fact that obese individuals present three times more risk of developing OA when compared to the ones with a healthy weight.^(^ ^8^ ^)^

Weight excess is considered to be a modifiable risk factor for OA.^(^ ^4^ ^)^ Body weight reduction has been recommended as an important component of OA treatment. There are reports of reduction in pain and physical disability in patients with OA and weight excess after moderate reduction in body weight.^(^ ^9^ ^,^ ^10^ ^)^ In addition to the mechanical overload produced by the excessive weight, there is a possible influence of adipocytokines^(^ ^11^ ^)^ and altered carbohydrate and lipid metabolism, that may generate characteristic changes of a chronic inflammatory state observed in these patients.^(^ ^12^ ^)^

A recent study compared obese and non-obese elderly individuals with knee OA based on the body mass index (BMI), and showed the obese ones presented less functional mobility, slower gait speed, higher pain intensity, and difficulty in performing daily living activities when compared to those with a healthy weight.^(^ ^13^ ^)^ Nonetheless, the sample was relatively small (n=35), and the authors recognized that a body composition assessment could bring more accurate results, since the isolated use of the BMI does not allow differentiation between fat and lean mass.^(^ ^14^ ^)^

Besides BMI, other more accurate ways to assess body composition may contribute to a better understanding about the impact of weight excess in OA. Since knowledge about the relation between weight excess and pain, especially in patients with OA, is limited,^(^ ^12^ ^)^ acknowledging and understanding the factors that may interfere in functional capacity and pain in individuals with OA may contribute to the development of novel treatment and prevention strategies of such prevalent disease.

## OBJECTIVE

To assess the influence of the body weight in functional capacity and pain of adult and elderly individuals with knee osteoarthritis.

## METHODS

This was an observational transversal study of 107 adult and elderly individuals with knee OA. The research was conducted between August 2013 and November 2014. Participants were recruited by means of a screening among patients referred to the physical therapy department of a private university in São Paulo (SP), Brazil. Phone contact was made to the patients who had diagnosis of OA, and those interested in participating in the research were invited to attend a session to receive information about the investigation. All patients received physical therapy treatment after participation.

Patients who met the following inclusion criteria were included in the study: diagnosis of OA, medical recommendation to participate in a physical therapy program, no chronic use of any medication (except the drugs prescribed for OA). Individuals with total or partial prosthesis in one or both knees or hips, decompensated hypertension or other heart diseases, rheumatoid arthritis, fibromyalgia or neurological disorders that could affect locomotion were excluded from the study.

Patients who agreed in participating the research underwent the following assessments: anthropometry, body composition, pain, joint stiffness and functionality, as well as pressure pain tolerance threshold.

The anthropometric evaluation consisted of measures of weight and height. Body weight (kg) was assessed with the participants barefoot and with light clothes, in a digital scale, graduated in 0.1kg. Height (cm) was measured with a stadiometer graduated in 0.1cm. Body mass index (kg/m^2^) was calculated from the weight and height, and participants were classified as having adequate body weight (BMI <25kg/m^2^) or excessive weight (BMI ≥25kg/m^2^).

Body composition was evaluated by electrical bioimpedance (Biodynamics BIA 450 with gel electrodes). Participants were instructed not to eat for 4 hours before the test, to drink plenty of water (especially 1 hour before the exam), not to drink alcoholic beverages or substances containing caffeine 48 hours prior to the test, and not to perform strenuous physical activities 24 hours before the exam. Prior to the examination, volunteers were instructed to empty the bladder and remove metallic objects from the body. Participants remained at rest for 10 minutes before the evaluation. The classification of results regarding adiposity (%F) was made in accordance with the American College of Sports Medicine (ACSM) recommendation,^(^ ^15^ ^)^ based on sex and age group, in adequate adiposity or excessive adiposity.

The Visual Analogue Scale (VAS)^(^ ^16^ ^)^ was used to assess pain intensity. To evaluate pain, joint stiffness and functionality, the Western Ontario and McMaster Universities Osteoarthritis Index (WOMAC)^(^ ^17^ ^)^ was employed in its Portuguese version.^(^ ^18^ ^)^ The WOMAC scores are presented in Likert scale, in which each question has a score ranging from zero to 100, distributed as follows: zero, if none; 25, little; 50, moderate; 75, severe; 100, very intense. Each of the dimensions (pain, stiffness and functionality) receives a score, transformed into a scale from zero (better health) to 100 points (worse health possible).

To measure functional mobility, the Timed Up and Go (TUG) test was used. It consists of measuring in seconds the time spent by the individual to get up from a chair, walk 3m, go back and sit again. The test was repeated three times and the shortest time obtained was selected for the analysis.^(^ ^19^ ^)^

Algometry was employed to evaluate the pressure pain tolerance threshold (J Tech Algometer, Salt Lake City, UT, United States). The algometer is a hand device formed by a piston that contains a 1cm diameter rubber in one end, which is capable of recording, through its electronic device, the pressure applied on a given surface. Its reliability was previously demonstrated.^(^ ^20^ ^,^ ^21^ ^)^ The pressure of 1kg/cm^2^ was applied at constant speed, at a 90° angle between the stimulation surface and the stimulated point, over all points to the extent the participant reported pain or discomfort. Reading was expressed in pounds and later converted into kilograms. During the evaluation, each participant was instructed to say “stop” as soon as the feeling of pressure went from unpleasant to painful. Some points in the vastus medialis and vastus lateralis muscles were assessed, as previously described.^(22)^

Data were analyzed using the Statistical Package for the Social Sciences (SPSS), version 22, for Windows, and the results were expressed as means±standard deviation. The distribution of the data was analyzed using D’Agostino Pearson test. Comparisons between groups (adequate weight/excessive weight; adequate adiposity/excessive adiposity) were made through Student’s *t* test, with significance level set at 5% (p<0.05).

The present study was approved by the Research Ethics Committee of ***Centro Universitário Adventista de São Paulo***, under protocol number 243,745, CAAE: 15199713.2.0000.5377. All research participants signed the informed consent form.

## RESULTS

The sample comprised 107 individuals with knee OA, predominantly females with mean age of 61.8±10.1 years (Table 1). Most participants (62.6%; n=67) had OA in both knees, 21.5% (n=23) only in the left knee and 15.9% (n=17) had OA in the right knee only.

**Table 1:** Demographic data

	n (%)	Mean±standard deviation	Median
Men	20 (18.7)		
Women	87 (81.3)		
Age		61.8±10.1	61.0
Height		157.8±6.9	158.5
Weight		78.8±13.3	77.6
BMI		31.7±5.5	31.4
%F		36.2±7.4	37.5

BMI: body mass index; %F: percent body fat.

Data on pain (duration, VAS and algometry) and functionality (TUG and WOMAC) of the total sample are shown in table 2.

**Table 2:** Functionality and pain in the whole sample

	Mean±standard deviation	Median
Duration of pain (months)	83.7±81.6	60.0
VAS	7.4±3.4	8.0
WOMAC - pain	59.3±17.8	60.0
WOMAC - stiffness	55.2±28.2	62.5
WOMAC - functionality	57.2±18.7	58.8
TUG	12.4±4.8	11.4
Algometry VM	3.0±1.7	2.9
Algometry VL	3.4±1.5	3.2

VAS: Visual Analogue Scale; WOMAC: Western Ontario and McMaster Universities Osteoarthritis Index; TUG: Timed Up and Go; VM: vastus medialis; VL: vastus lateralis.

When dividing the group by BMI (<25 and ≥25) and by %F (ACSM, 2014),^(^ ^15^ ^)^ in both cases, 89.7% of sample (n=96) had excessive weight/excessive adiposity. The predominance of women was significantly higher in the group with BMI ≥25 than in the group with BMI <25 (p=0.016). The same was observed for the adiposity groups: there was significantly more women in the group with excessive adiposity than the in group with adequate adiposity (p=0.001).

The group with BMI <25 had a mean BMI of 23.5±1.8, whereas in the group of patients with BMI ≥25, the mean BMI was 32.6±4.9. When divided by adiposity, the group that presented adequate adiposity had a mean BMI of 25.1±2.8, and in the group with excessive adiposity, the mean BMI was 32.5±5.2. Comparison between groups revealed that both the heavier BMI and those with higher adiposity (%F) presented significantly more pain when evaluated by WOMAC. However, there was no difference between groups regarding duration of pain, pain assessed by VAS, algometry and functionality (Table 3).

**Table 3:** Comparison of functionality and pain between patients with and without excessive weight and excessive adiposity

	BMI <25 (n=11)	BMI ≥25 (n=96)	Adequate adiposity (n=11)	Excessive adiposity (n=96)
Age (years)	60.9±10.7	61.9±10.1	65.5±12.4	61.4±9.8
Duration of pain (months)	103.1±95.1	81.5±80.3	79.2±92.4	84.3±80.8
VAS	5.9±3.4	7.5±3.3	5.2±2.8	7.6±3.3
WOMAC - pain	47.3±25.0	60.7±6.4*	53.6±25.6	59.9±16.8*
WOMAC - stiffness	51.1±29.8	55.6±28.2	57.9±37.6	54.8±27.2
WOMAC - functionality	42.9±22.7	58.8±17.6	46.8±22.3	58.4±17.9
TUG	12.1±4.7	12.4 ±4.8	11.8±5.8	12.5±4.6
Algometry VM	3.9±1.7	2.9 ±1.7	4.1±1.7	2.9±1.7
Algometry VL	3.5±1.2	3.4±1.6	4.0±1.5	3.3±1.5

Data expressed as means±standard deviations; *p=0.05 between groups. BMI: body mass index; VAS: Visual Analogue Scale; WOMAC: Western Ontario and McMaster Universities Osteoarthritis Index; TUG: Timed Up and Go; VM: vastus medialis; VL: vastus lateralis.

Patients were also classified as obese (BMI <30kg/m^2^) and non-obese (BMI ≥30kg/m^2^), as shown in table 4. This analysis showed that patients with OA and obesity present significantly greater pain perception, assessed by VAS (p=0.05) than the non-obese ones.

**Table 4:** Comparison of functionality and pain between obese and non-obese patients

	BMI <30 (n=43)	BMI ≥30 (n=64)
Age (years)	62.9±11.6	61.1±9.0
Duration of pain (months)	85.1±88.9	82.8±77.4
VAS	6.4±3.0	7.6±2.3*
WOMAC - pain	58.5±20.1	60.2±15.3
WOMAC - stiffness	58.6±28.5	53.2±27.5
WOMAC - functionality	52.5±19.6	60.8±16.7
TUG	12.2±5.0	12.5±4.6
Algometry VM	3.2±1.7	2.9±1.7
Algometry VL	3.5±1.4	3.3±1.6

Data expressed as means ± standard deviations. *p=0.009 between groups. BMI: body mass index; VAS: Visual Analogue Scale; WOMAC: Western Ontario and McMaster Universities Osteoarthritis Index; TUG: Timed Up and Go; VM: vastus medialis; VL: vastus lateralis.

## DISCUSSION

In both forms of classification used in this study (BMI and adiposity), individuals with OA who presented excessive weight or excessive adiposity differed in WOMAC pain results in relation to those who presented adequate weight and adiposity.

The literature reports that obese individuals are likely to overload their joints more and, therefore, have greater disabilities.^(^ ^23^ ^)^ However, contrary to this assumption and to the findings by Gomes-Neto et al.,^(^ ^13^ ^)^ (who verified greater pain and disability in patients with BMI>25), the sample of the present study showed differences only in pain assessed by WOMAC, but none in functionality and stiffness evaluated by the same instrument. When classified as obese (BMI ≥30 and non-obese (BMI <30), groups were different regarding pain perception only (VAS).

In addition to BMI, excessive adiposity may also be related to increased pain sensitivity,^(^ ^24^ ^)^ however, despite the lower absolute tolerance observed in individuals with excessive weight/excessive adiposity, no statistically significant differences were found between groups. This probably reflects the fact that the increase in body weight is related to the increase in pain.^(^ ^25^ ^)^

Regarding the other forms of pain assessment employed in this study, although values of VAS and algometry were worse in the excessive weight/excessive adiposity or obese groups and better in the groups with adequate weight/adiposity and non-obese patients, no significant differences were found between them. In our sample, WOMAC was a more sensitive instrument to assess pain when weight or adiposity excess was the criteria to split groups. However, VAS was efficient in discriminating pain perception when patients were classified as obese or non-obese.

Although pain intensity may influence the performance of functional activities in obese individuals with knee OA,^(^ ^26^ ^)^ in this study, unexpectedly, there was no difference between groups in the TUG test, which evaluates functional mobility by means of an activity involving standing up, walking and sitting down.

When comparing women with bilateral knee OA (13 obese, BMI >30kg/m^2^ and 15 with morbid obesity, BMI >40kg/m^2^), Vasconcelos et al.,^(^ ^27^ ^)^ found that the degree of obesity had no impact on the symptoms of pain, stiffness and functional difficulties (evaluated by WOMAC). The authors concluded that other factors should influence functional performance of obese women with knee OA. This may explain the fact that we found no difference in the time to perform TUG between the groups of this study. The mean differences of 0.3 seconds found between groups when separated by BMI (≥25 or ≥30), and 0.7 seconds when separated by adiposity, were not significant but show that these individuals probably have already experienced functional mobility problems, since their mean time to perform the test was higher than 10 seconds.^(^ ^19^ ^)^ However, a previous study^(^ ^28^ ^)^ showed even higher values in TUG of individuals with knee OA, and BMI similar to those of the patients in this study.

In the present study, besides using BMI as a classification criterion, as already done by other authors,^(^ ^13^ ^)^ we employed a classification that portrayed the body composition of lean mass and fat mass of the participants, and not only the presence of alterations in the individual’s energy balance.^(^ ^29^ ^)^ However, in the sample evaluated, the BMI was an acceptable tool to estimate adiposity, since the classification of the participants by BMI (< or ≥25) resulted in data very similar to those obtained when categorizing by adiposity (adequate or excessive).

It should also be noted that, in this study, weight or adiposity excess were very prevalent conditions in patients with knee OA. In the group of 107 patients studied, 89.7% presented excess of weight or adiposity when evaluated by BMI or %F, respectively, and 59.8% of them were obese (BMI ≥30). It is known that obesity, besides being widely recognized as a risk factor for OA, contributes to enhance severity of the disease.^(^ ^29^ ^)^ This highlights the magnitude of the problem represented by excessive weight in OA patients, besides raising and reinforcing the thesis that only therapeutic exercises may not be fully effective for treating the disease. Likewise, the control of body weight and adiposity, education about the disease, cognitive-behavioral therapy, among other approaches, seem to be associated with a better management of chronic diseases, such as OA.^(^ ^30^ ^)^

Future research with even more accurate methods of body composition assessment, such as computed tomography, can confirm the results of the present study, as well as establish relations between body composition and other variables that may influence the quality of life and functionality of individuals with knee OA.

Participants from this study were recruited among individuals referred to a physical therapy department, and their weight and adiposity were evaluated along with other assessments prior to referral to treatment. All volunteers had diagnosis of OA, but no classification of the degree of the disease, which limits our conclusions. Although this form of selection has resulted in discrepant number of participants in both groups, this distribution probably reflects the actual condition of the body composition in patients with OA in the daily medical and rehabilitation practices. The low number of participants in the groups with adequate BMI and %F may have prevented the observation of some significant differences between groups. Despite these limitations, our results provide important new pieces of information on the influence of body weight and body composition in individuals with knee OA.

## CONCLUSION

Weight and adiposity excess or obesity had a negative impact in patients with osteoarthritis, increasing their pain perception. Although mean results of functional capacity and pressure pain tolerance were worse in patients with weight excess or adiposity excess, differences in these parameters did not reach statistical significance when compared with the adequate weight or adiposity groups.

## References

[1] Allen KD, Golightly YM (2015). State of the evidence. Curr Opin Rheumatol.

[2] Alkan BM, Fidan F, Tosun A, Ardıçoğlu O (2014). Quality of life and self-reported disability in patients with knee osteoarthritis. Mod Rheumatol.

[3] Zhang Y, Jordan JM (2010). Epidemiology of osteoarthritis. Clin Geriatr Med.

[4] Sofat N, Ejindu V, Kiely P (2011). What makes osteoarthritis painful? The evidence for local and central pain processing. Rheumatology (Oxford).

[5] Hunter DJ, McDougall JJ, Keefe FJ (2008). The symptoms of osteoarthritis and the genesis of pain. Rheum Dis Clin North Am.

[6] Salaffi F, Ciapetti A, Carotti M (2014). The sources of pain in osteoarthritis: a pathophysiological review. Reumatismo.

[7] Radominski SC (1998). Obesity and musculoeskeletal diseases. Rev Bras Reumatol.

[8] Blagojevic M, Jinks C, Jeffery A, Jordan KP (2010). Risk factors for onset of osteoarthritis of the knee in older adults: a systematic review and meta-analysis. Osteoarthritis Cartilage.

[9] Christensen R, Bartels EM, Astrup A, Bliddal H (2007). Effect of weight reduction in obese patients diagnosed with knee osteoarthritis: a systematic review and meta-analysis. Ann Rheum Dis.

[10] Jinks C, Jordan K, Croft P (2006). Disabling knee pain -- another consequence of obesity: results from a prospective cohort study. BMC Public Health.

[11] Presle N, Pottie P, Dumond H, Guillaume C, Lapicque F, Pallu S (2006). Differential distribution of adipokines between serum and synovial fluid in patients with osteoarthritis. Contribution of joint tissues to their articular production. Osteoarthritis Cartilage.

[12] Sowers MR, Karvonen-Gutierrez CA (2010). The evolving role of obesity in knee osteoarthritis. Curr Opin Rheumatol.

[13] Gomes M, Araujo AD, Junqueira ID, Oliveira D, Brasileiro A, Arcanjo FL (2016). Comparative study of functional capacity and quality of life among obese and non-obese elderly people with knee osteoarthritis. Rev Bras Reumatol Engl Ed.

[14] Cortez AC, Martins MC (2012). Anthropometric indicators of nutritional status in elderly: as system review. UNOPAR Cient Cienc Biol Saude.

[15] American College of Sports Medicine (ACSM) (2014). Guidelines for exercise testing and prescription.

[16] Chapman RS, Syrjala KL, Bonica JJ (1990). Measurement of pain. The management of pain.

[17] Bellamy N, Buchanan WW, Goldsmith CH, Campbell J, Stitt LW (1988). Validation study of WOMAC: a health status instrument for measuring clinically important patient relevant outcomes to antirheumatic drug therapy in patients with osteoarthritis of the hip or knee. J Rheumatol.

[18] Fernandes MI (2001). Tradução e validação do questionário de qualidade de vida específico para osteoartrose WOMAC (Western Ontario and McMaster Universities) para a língua portuguesa.

[19] Podsiadlo D, Richardson S (1991). The timed “Up & Go”: a test of basic functional mobility for frail elderly persons. J Am Geriatr Soc.

[20] Ylinen J, Nykänen M, Kautianine H, Häkkinen A (2007). Evaluation of repeatability of pressure algometry on the neck muscles for clinical use. Man Ther.

[21] Visscher C, Lobbezoo F, Naeije M (2004). Comparison of algometry and palpation in the recognition of temporomandibular disorder pain complaints. J Orofac Pain.

[22] Imamura M, Imamura ST, Kaziyama HH, Targino RA, Hsing WT, de Souza LP (2008). Impact of nervous system hyperalgesia on pain, disability, and quality of life in patients with knee osteoarthritis: a controlled analysis. Arthritis Rheum.

[23] Rodrigues Franco L, Santos Simão L, Oliveira Pires ED, Alves Guimarães E (2009). Influence of age and obesity in the diagnosis of osteoarthritis of knee. ConScientiae Saude.

[24] Khimich S (1997). Level of sensitivity of pain in patients with obesity. Acta Chir Hung.

[25] Heo M, Allison DB, Faith MS, Zhu S, Fontaine KR (2003). Obesity and quality of life: mediating effects of pain and comorbidities. Obes Res.

[26] Vasconcelos KS, Dias JM, Dias RC (2006). Relationship between pain intensity and functional capacity individuals with knee osteoarthritis. Rev Bras Fisioter.

[27] Vasconcelos KS, Dias JM, Dias RC (2008). Impact of the degree of obesity on symptoms and functional capacity of women with knee osteoarthritis. Fisioter Pesq.

[28] Arthur K, Nascimento LC, Figueiredo DA, Souza LB, Alfieri FM (2012). Effects of geotherapy and phytitherapy associated with kinesiotherapy in the knee osteoarthritis: randomized double blind study. Acta Fisiatr.

[29] Bliddal H, Leeds AR, Christensen R (2014). Osteoarthritis, obesity and weight loss: evidence, hypotheses and horizons - a scoping review. Obes Rev.

[30] Ambrose KR, Golightly YM (2015). Physical exercise as non-pharmacological treatment of chronic pain: Why and when. Best Pract Res Clin Rheumatol.

